# Post–lung transplant surveillance in 2026: current practice, variability, and the need for standardization

**DOI:** 10.3389/ti.2026.16295

**Published:** 2026-05-13

**Authors:** Berta Saez-Gimenez, Jesper M. Magnusson, Andrea Zajacova

**Affiliations:** 1 Respiratory Department, Hospital Universitari Vall d’Hebron, Barcelona, Spain; 2 Department of Pulmonology, Sahlgrenska University Hospital, Gothenburg, Sweden; 3 Transplant Institute, Gothenburg, Sweden; 4 Laboratory of Respiratory Diseases and Thoracic Surgery (BREATHE), Department CHROMETA, KU Leuven, Leuven, Belgium

**Keywords:** follow-up, graft survival, lung transplantation, outcome, surveillance

## Abstract

Post–lung transplant surveillance remains highly heterogeneous, with no universally accepted standard guiding organisation of care or the use of physiological testing, imaging, bronchoscopy, laboratory monitoring, and emerging biomarkers. This narrative review synthesises current surveillance practices across these domains and addresses key limitations, sources of inter-centre variability, and evidence gaps that hinder timely detection of allograft dysfunction. We summarize established and evolving approaches to organisation of care, lung function monitoring, radiological assessment, invasive diagnostics, and laboratory parameters, along with novel biomarkers, highlighting where evidence supports routine use and where tools remain investigational. Fragmentation of follow-up strategies, inconsistent interpretation of longitudinal data, and limited integration of novel diagnostics contribute to delayed recognition of graft injury and variable outcomes. Advancing post-transplant care will require consensus-driven definition of minimum surveillance standards, trajectory-based interpretation frameworks, and rational incorporation of validated biomarkers and digital technologies into harmonised follow-up pathways.

## Introduction

**FIGURE 1:**
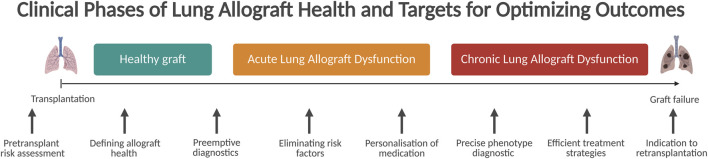
Clinical phases of lung allograft health and targets for optimizing outcomes.

Post–lung transplant (LTx) follow-up is complex, multidisciplinary, and highly variable across centres, with no universally adopted standard. Differences in the use of pulmonary function testing, imaging, laboratory testing, bronchoscopy, and emerging technologies result in substantial heterogeneity in clinical practice. In this narrative review, we summarise the current state-of-the-art approaches to LTx surveillance and highlight areas where practice varies and further harmonisation is needed. To inform this narrative review, we performed targeted PubMed searches between October 2025 and January 2026, focusing on available tools to monitor the graft after lung transplantation. Figure 1 provides a conceptual overview of the clinical trajectory from transplantation to graft failure, highlighting the key surveillance objectives that structure this review. The primary aim of our review is to highlight the existing variability in clinical practice and to identify the current gaps in standardization across surveillance strategies.

## Post-lung transplant follow-up care organisation

### The transplant pulmonologist as the central coordinator of care

LTx care is inherently a respiratory discipline and therefore must be anchored in specialised pulmonological follow-up. Transplant pulmonologists build on core respiratory training with dedicated expertise in lung allograft physiology, chest imaging, lung allograft-specific diagnostics including bronchoscopy, bronchoalveolar lavage (BAL), transbronchial biopsies (TBB), advanced lung-function interpretation, and chronic lung allograft dysfunction (CLAD) phenotyping. Because most post-LTx complications present with respiratory abnormalities, early recognition and effective management require specialised pulmonary expertise. While multidisciplinary care is essential, no other specialty integrates respiratory physiology, imaging, and procedural assessment throughout long-term follow-up. This central coordinating role is particularly critical when integrating multimodal surveillance data and resolving discrepant findings (e.g., discordance between imaging, bronchoscopy, and emerging biomarkers), where expert clinical judgment is required to guide management.

**FIGURE 2:**
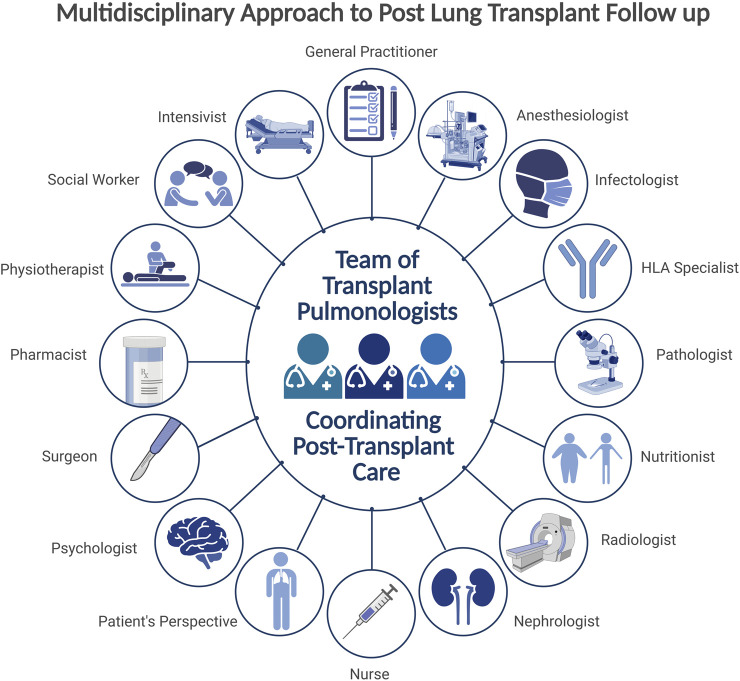
Central role of the transplant pulmonologist within a multidisciplinary post-transplant care team.

General pulmonologists may also contribute meaningfully—especially for patients distant from transplant centres—but optimal outcomes require a specialized transplant pulmonologist-coordinated care model [1] Figure 2.

### Site of follow-up

Long-term surveillance is highly specialised, reflecting the interplay between complex allograft biology and substantial burden of extra-pulmonary comorbidities, and therefore requires coordinated multidisciplinary care within experienced transplant programmes [2]. LTx recipients frequently present with significant baseline multimorbidity—including cardiovascular, metabolic, and psychiatric conditions—which independently affects mortality and necessitates systematic assessment and follow-up [3, 4]. In parallel, increasing recognition of genetic and phenotypic heterogeneity in fibrotic lung disease underscores the need for centres capable of integrating molecular diagnostics, genetic screening, and precision-medicine approaches into post-transplant care [5–7].

Where follow-up is delivered also influences outcomes. In cystic fibrosis (CF), transplantation at centres hosting accredited CF programmes is associated with an approximately 33% lower risk of graft failure or death compared with non-CF centres, independent of centre volume [8]. More broadly, matched registry analyses demonstrate superior 5-year survival in European centres compared with North America despite similar early outcomes, suggesting that organisational structures and/or better access to long-term care meaningfully affect prognosis [9]. Advanced practice providers (nurse practitioners, physician assistants) are increasingly embedded within LTx programmes to enhance continuity, coordinate routine surveillance, and comorbidity management, and serve as a liaison between transplant centres and local clinicians [10, 11].

### Documentation and monitoring

Comprehensive and longitudinal documentation is essential for qualitative post-LTx follow-up. Fragmentation inherent to paper-based records has driven adoption of electronic logbooks and transplant registries that consolidate clinical visits, investigations, and interventions. These systems facilitate audit, enable linkage with national registries, and support quality-improvement initiatives [12, 13]. From the patient perspective, personal logbooks—physical or digital—promote engagement and awareness of key health metrics, while integration of electronic alerts for test scheduling and abnormal results further strengthens safety in long-term transplant care [14, 15].

### Patient-reported outcomes and experience measurements (PROMs and PREMs)

Structured post-LTx follow-up integrates subjective and objective assessment. Subjective evaluation captures patient-reported symptoms, medication side effects, and psychosocial wellbeing and can be standardised through validated PROMs, several of which are established in LTx [2, 16–18]. PROMs extend beyond conventional clinical data by capturing health status and quality of life, including symptoms, functional status, and psychological wellbeing [12, 19, 20]. PREMs complement PROMs by assessing patients’ experience of care, including access, communication, coordination, and shared decision-making [21].

Both tools can be integrated into clinical workflows through digital pre-visit questionnaires or tablet-based assessments, enabling real-time identification of patient needs, individualisation of care, and longitudinal monitoring. Standardised use of PROMs and PREMs also facilitates benchmarking across transplant centres and supports quality improvement initiatives [22].

### Role of telemedicine

Telemedicine is increasingly used to extend specialist follow-up after LTx. Telemonitoring, teleconsultation, telerehabilitation, and telespirometry can enhance surveillance, support patient-centred care, and promote self-management [23]. The INSPIRE-III randomised trial demonstrated that telehealth-delivered coping skills and exercise interventions are feasible after LTx and provide modest benefits in psychological distress and functional capacity, particularly among patients with baseline depression [24]. Observational studies report high patient satisfaction and reduced travel burden, although most patients prefer periodic in-person visits for complex assessments [15, 19, 25, 26]. Collectively, available evidence supports a hybrid care model in which regular in-person follow-up at specialised centres is augmented—but not replaced—by structured telemedicine.

Telemedicine may also support assessment of treatment adherence by enabling earlier detection of missed doses or behavioural barriers. As non-adherence is a major determinant of graft survival [20, 27, 28], integrating remote monitoring with in-person care may facilitate earlier intervention. Nevertheless, in-person visits remain essential for adherence reinforcement through pharmacy refill checks, drug-level variability indices, and validated self-report tools such as BAASIS [27–31].

## Pulmonary function testing

### Spirometry

Spirometry remains the cornerstone of physiological allograft monitoring, with forced expiratory volume in 1 second (FEV_1_) serving as the basis for detecting and following lung allograft dysfunction. Percent-predicted thresholds are derived from healthy populations measurements, nevertheless, in complex post-transplant setting—heavily affected by size matching, thoracic wall disturbances (caused by both primary diagnosis and surgical approaches), respiratory muscle weakness, or receiving unilateral or lobar transplant—percent-predicted values are often misleading in assessing normality [32–37]. Accordingly, International Society for Heart and Lung Transplantation (ISHLT) guidelines define CLAD based on relative decline from an individualised post-transplant baseline using absolute FEV_1_ values rather than population norms [38]. To contextualise lung function with standard population-based norms, the term baseline lung allograft dysfunction (BLAD) has been proposed to describe recipients who never achieve expected predicted values, awaiting the publication of a consensus document from the ISHLT [39–41].

Spirometry interpretation is further complicated by historical drift from the original Tiffeneau–Pinelli index (FEV_1_/slow vital capacity; SVC) toward the forced expiratory ratio (FEV_1_/forced vital capacity; FVC). FVC may be underestimated during forceful expiration due to dynamic airway collapse, particularly in bronchiolitis obliterans syndrome (BOS), thereby masking airflow obstruction [42, 43]. In contrast, SVC—measured during slow exhalation—more accurately reflects true vital capacity and minimises this artefact, as demonstrated in multiple studies [44–46]. Despite this, routine SVC measurement requires additional time, technical expertise, and transplant-specific reference values and current CLAD definitions therefore rely on FEV_1_/FVC, which should be interpreted cautiously and contextualised (i.e., with flow–volume loops) [38, 47].

Machine learning (ML) introduces novel analytical approaches to spirometry, with preliminary data suggesting potential utility for CLAD prediction [48]. Digital home spirometry enables frequent remote monitoring with reliable FEV_1_ measurements that correlate well with laboratory values and may improve early detection of lung function decline [35–37].

### Body plethysmography

In post-LTx clinical monitoring, plethysmography has prognostic value and is required for establishing total lung capacity (TLC) baseline (at 6 and 12 months post-transplant) and for restrictive CLAD diagnosis and phenotyping, including restrictive allograft syndrome (RAS) and "mixed/undefined phenotypes" [38]. Despite this, a recent comprehensive European survey revealed that approximately 16% of centres managing CLAD do not routinely use this method [49]. Another limitation occurs in patients with severe dyspnoea, who may be unable to perform the panting manoeuvre, leading to discomfort and unreliable resistance and volume measurements [50]. Consequently, reliable plethysmographic TLC measurement in advanced CLAD can be challenging, underscoring that TLC assessment is most informative during stable follow-up and early CLAD phenotyping. While the optimal measurement interval is not well established, extending intervals relative to standard spirometry may help mitigate cost and availability constraints.

### Lung diffusion capacity testing

Reduced diffusing capacity of the lung for carbon monoxide (DLCO) has independent prognostic value for survival, with declines associated with increased mortality even after adjustment for FEV_1_ and FVC [51]. This suggests DLCO captures aspects of allograft health not reflected by spirometry or body plethysmography, likely related to gas exchange and pulmonary vascular integrity. DLCO is well-established for monitoring disease progression in interstitial lung diseases, where abnormalities often precede spirometric changes [52, 53], supporting its complementary role among pulmonary function tests, despite not being fully integrated into current post-transplant guidelines. Although TLC can be estimated using the single-breath helium dilution manoeuvre during DLCO testing, this method underestimates true TLC in the presence of ventilation inhomogeneity and is therefore less reliable than plethysmography [54].

### Novel physiological methodology in lung transplant follow-up

Oscillometry measures distal airway resistance during tidal breathing and might be more sensitive than spirometry for detecting early rejection, although longitudinal data remain limited [38, 55–58]. Because it relies on normal tidal breathing, oscillometry is particularly useful in patients unable to perform forced manoeuvres reliably, including children and frail individuals.

Fractional exhaled nitric oxide provides a rapid, non-invasive marker of airway inflammation and is elevated in infection, lymphocytic bronchiolitis, and acute rejection. However, despite high sensitivity, it lacks specificity and does not predict functional decline, limiting its role to supportive use during routine visits [59–63].

Lung clearance index, obtained by multiple-breath washout, detects ventilation heterogeneity and is highly sensitive to small-airway dysfunction, often preceding spirometric changes [63, 64]. Evidence is strongest in paediatric recipients, while adult data remain limited [65, 66].

### Novel biomarkers

Exhaled breath condensate analysis combined with ML has shown promise in discriminating between different forms of lung allograft dysfunction, however, further validation is required, although the practicality and rapidity of electronic-nose platforms suggest potential as a point-of-care diagnostic tool [67, 68].

## Imaging

### Chest X-ray

Chest X-ray remains an essential first-line screening tool after LTx due to its rapid availability, low radiation exposure, and utility in detecting primary graft dysfunction and other post-transplant complications, including pneumothorax, pleural effusions, or pneumonia [69]. Acquisition of both posteroanterior and lateral projections is critical, as the lateral view improves detection of subtle pleural, parenchymal, and mediastinal abnormalities that may be missed on a single-plane projection [70].

### Computed tomography (CT)

In contemporary practice, emphasis is better placed on CT protocol design than on older nomenclature such as high-resolution CT, because modern multidetector scanners generally provide high-resolution volumetric data. The clinically relevant distinction is whether appropriate non-contrast inspiratory and expiratory acquisitions are obtained, ideally using dose-optimized techniques [71]. Expiratory scans are particularly important, as it reveals air trapping—a hallmark feature of BOS—and frequently detects dysfunction earlier than inspiratory imaging [71–73]. However, its diagnostic utility depends strongly on acquisition quality, and inadequate expiratory effort can render studies non-diagnostic [71]. Standard CT retains a complementary role, particularly for assessing complications such as tree-in-bud, ground glass opacities, (sub)pleural consolidations, pulmonary embolism, vascular stenosis, large-airway pathology, or mediastinal abnormalities [73].

#### Surveillance CT

An initial baseline CT with both inspiratory and expiratory scans with a maximum width of 3-mm sections at 6 months post-LTx is recommended to enable a baseline for future comparison [38]. Among CT features, air-trapping is one of the most informative markers of BOS, outperforming mosaic perfusion, bronchiectasis, and bronchial wall thickening, although reported sensitivity varies [72, 74, 75]. Lung consolidations and pleural effusions have also been linked to subsequent BOS development and reduced survival [75].

Quantitative CT (qCT) techniques further enhance surveillance. Voxel-wise density mapping of paired inspiratory and expiratory scans at 6 months post-LTx outperformed conventional expiratory threshold-based methods and correlated with residual volume/TLC [76]. Longitudinal parametric response mapping links radiographic small-airway disease with peri-BOS FEV_1_ decline, supporting its adjunctive prognostic value [77]. A consensus on the minimum frequency of imaging required for routine graft surveillance after LTx is still lacking.

#### Diagnostic CT

Beyond routine surveillance, CT is essential when new respiratory symptoms or lung-function decline raise suspicion of acute or chronic allograft dysfunction, as it aids in identifing underlying causes such as infection, acute rejection, CLAD, or malignancy. Several reviews provide detailed guidance on post-LTx radiologic assessment and CLAD imaging patterns [78, 79].

In unilateral LTx recipients with a ≥10% FEV_1_ decline, combining pulmonary function testing with qCT metrics using supervised ML algorithms improved BOS detection compared with spirometry alone [80] and helped distinguish BOS from non-BOS causes of FEV_1_ decline [81]. Among patients with a 10%–20% FEV_1_ reduction, the presence of any abnormal parametric response mapping signature—functional small airway disease or parenchymal disease—was associated with shorter CLAD-free survival [82].

Although imaging contributes to CLAD subclassification, current ISHLT recommendations do not specify precise follow-up CT protocol that should be used [38]. Nevertheless, CT with paired inspiratory-expiratory scans could therefore be considered for all patients with suspected CLAD because of its phenotyping and prognostic value, and most European centres routinely acquire both inspiratory and expiratory scans at CLAD suspicion [49]. While air-trapping is frequently identified, it did not predict CLAD-free survival in a BOS cohort; in contrast, ground-glass opacities were associated with better outcomes, with deep neural networks demonstrating strong performance in BOS prediction [83]. Conversely, bronchiectasis, peribronchial thickening, and parenchymal changes were linked to inferior survival in BOS [84]. In RAS, prognostic interpretation of CT with expiratory scans remains heterogeneous, with some studies reporting no outcome correlation and others demonstrating prognostic value using inflammation-based scoring systems [85, 86]. qCT techniques—including texture analysis and CT density histograms—improve phenotyping and often predict BOS, RAS, or mixed phenotypes earlier, with clearer survival implications [87–89]. ML–enhanced radiology further refines prognostication; features such as reticulation and pulmonary vessel volume show strong diagnostic and prognostic performance, with pulmonary vessel volume emerging as a particular robust biomarker [90]. Although no formal guidelines define radiologic follow-up in established CLAD, serial CTs with expiratory scans may be appropriate in selected cases to assess phenotype transition from BOS to RAS, which may carry prognostic significance [91, 92].

### Magnetic resonance

MRI is an emerging imaging tool in post-LTx follow-up. Hyperpolarised gas MRI—particularly ^3^He-MRI—can detect early regional ventilation abnormalities in BOS but remains limited by cost, ^3^He availability, and the need for specialised equipment [93, 94]. Consequently, more feasible non-hyperpolarised MRI techniques have gained interest. Oxygen transfer function MRI demonstrates reduced values in BOS [95], while functional approaches such as Fourier decomposition and phase-resolved functional lung (PREFUL) MRI enable assesment of regional ventilation without contrast agents. In a cohort a cohort of 141 recipients, PREFUL ventilation metrics, including reduced relative fractional ventilation, predicted retransplantation or CLAD-related death, whereas FEV_1_ did not, with consistent threshold values reported across studies [96, 97]. Dynamic ^19^F MRI has similarly demonstrated quantifiable regional ventilation differences between CLAD and non-CLAD recipients, particularly in regional lung clearance indices, with strong correlations between peripheral ventilation and FEV_1_ [98].

### Complementary imaging: ultrasound, PET–CT, and perfusion scintigraphy

Chest ultrasound after LTx is primarily used for bedside assessment of pleural effusions, diaphragmatic motion, and procedural guidance for thoracentesis [99]. Nuclear imaging provides complementary information: PET–CT can help distinguish infection, malignancy, and inflammation, and support evaluation of suspected RAS by identifying metabolically active fibrotic or inflammatory regions [73, 100]. Perfusion scintigraphy remains useful when pulmonary vascular disease is suspected, aiding differentiation between vascular causes of functional decline and CLAD-related pathology [101], and may also support early BOS detection in single LTx recipients [102].

### Novel biomarkers

Emerging molecular imaging with fibroblast activation protein–targeted tracers offers the potential to non-invasively visualize fibrotic remodelling in lung allografts, providing a “biological” radiologic biomarker that may enable earlier detection and phenotyping of CLAD, although its use remains largely investigational [103].

## Bronchoscopy

### Follow-up protocols

Bronchoscopy with BAL and TBB remains the current gold standard for both surveillance and for-cause evaluation after LTx, enabling detection, confirmation, or exclusion of subclinical infection, rejection, or anastomotic complications, although no consensus exists regarding optimal surveillance intervals.

Comparisons between surveillance and for-cause bronchoscopy have yielded mixed results. Current evidence does not demonstrate that surveillance bronchoscopies improve survival or reduce CLAD, although most centres still perform them to detect subclinical rejection [104]. Interpretation across studies is limited by inconsistent definitions of “for-cause” bronchoscopy, typically triggered by FEV_1_ decline, new symptoms, hypoxaemia, or new radiological infiltrates. A recent meta-analysis including three small observational cohorts highlighted substantial heterogeneity in clinically indicated bronchoscopy use [105]. Detection rates for acute rejection were similar overall, although for-cause procedures yielded higher rates of grade A2–A4 acute cellular (ACR) rejection. Surveillance bronchoscopy nonetheless prompts therapeutic changes in 7%–31% of cases [106–108].

The goal of bronchoscopy strategies is to maximise diagnostic yield while minimising risk. Benefits include identification of infection and anastomotic complications and TBB remains the gold standard for diagnosing ACR and antibody-mediated rejection (AMR). These advantages must be balanced against procedure-related risks, including pneumothorax (0.1%–2%) and bleeding (1.9%–13%) [107, 109–111], mostly biopsy-related and more frequent in LTx recipients [112].

### Transbronchial biopsy

TBB is a cornerstone for definitive histologic diagnosis of graft injury/rejection after LTx, but it is limited by procedural risk, inadequate tissue yield, interobserver variability, and patient discomfort. Diagnostic sensitivity remains uncertain: early studies reported high sensitivity with extensive sampling [113], whereas modern practice (typically ≤5 samples) achieves only ∼30% sensitivity for acute rejection in both surveillance and for-cause procedures [107, 114, 115]. Interobserver agreement among pathologists is moderate to high for A-grade and fair for B-grade ACR [116, 117].

Surveillance TBB is supported by association between ACR or lymphocytic bronchiolitis and subsequent CLAD development [118–121], although correlations with overall survival are inconsistent [121, 122]. TBB has limited utility for CLAD detection [109] and it is not necessary for CLAD diagnosis [38]. Its diagnostic yield is highest within the first 12–16 weeks post-LTx, after which clinically significant findings decrease [106, 108].

Cryobiopsy remains investigational. A randomised study suggests comparable safety and diagnostic performance to forceps TBB, with higher specimen adequacy [123], and limited evidence indicates that four cryobiopsies may offer the best balance between diagnostic yield and safety [124], although the optimal number of samples required for ACR diagnosis remains undefined.

Endobronchial biopsies are an emerging, less invasive alternative to TBB, offering reproducible histologic and molecular markers of airway inflammation, ACR, and CLAD with a favourable safety profile, and may gain importance as molecular diagnostics evolve [125]. Additionally, airway brushing offers a minimally invasive means of sampling airway epithelial biology and has been used for molecular profiling of allograft injury—particularly CLAD—though its role in routine rejection surveillance remains investigational [126, 127].

### Bronchoalveolar lavage

BAL samples the small airways and alveolar compartment, and remains essential for diagnosing infectious complications, a key contributor to CLAD development [16, 128]. The ISHLT published a consensus statement in 2020, standardising BAL technique in LTx [104]. Observational studies show that surveillance BAL detects asymptomatic infections in 12%–40% of procedures, while for-cause BAL identifies pathogens in 39%–50% of cases [129, 130]. However, despite higher detection rates, surveillance BAL has not been shown to improve CLAD-free survival or overall survival. An ISHLT survey reported universal bacterial cultures of BAL fluid, with most centres also performing fungal (89%), mycobacterial (86%), and non-cytomegalovirus (CMV) viral polymerase chain reaction testing (70.2%) [104].

### Immunoprofiling

BAL lymphocyte differential profiles can support clinical interpretation of allograft status but are not diagnostic. In the ISHLT survey, 61% of centres routinely requested BAL cytology—most commonly for suspected infection (23%), malignancy (35%), or rejection (13%)—yet evidence remains insufficient to define diagnostic accuracy [104]. Assessment of cellular composition is performed in 72% of centres, and several studies have shown that BAL neutrophilia (cut-off ∼15–20%) is associated with both concurrent and future CLAD, even in patients receiving azithromycin [131, 132]. Elevated BAL eosinophilia (1%–2%) has similarly been linked to reduced CLAD-free survival, particularly in RAS [106, 133–135]. Accordingly, ISHLT consensus recommends inclusion of differential cell counts in all post-transplant BAL samples [104].

### Novel biomarkers

BAL has been extensively explored for LTx monitoring, including donor-derived cell-free DNA (dd-cfDNA), inflammatory cytokines and chemokines (e.g., IL-8, CXCL9–11), and extracellular vesicles or exosomes, yet none have been adopted into routine clinical practice [136–139]. In parallel, transcriptomic profiling of BAL cells is emerging as a tool for allograft assessment and tissue-based transcriptomics (already standard-of-care in kidney and heart transplantation) may offer enhanced precision and identify subclinical but remains investigational and not yet approved for LTx [140–146].

## Blood monitoring

### Therapeutic drug monitoring

Most recipients are maintained on calcineurin inhibitor (CNI)–based regimens combined with an antimetabolite and corticosteroids [2, 147]. CNI have a narrow therapeutic index with substantial intra- and interindividual pharmacokinetic variability, making routine therapeutic drug monitoring (TDM) essential [147]. CNI trough levels—measured immediately before the next dose—remain the standard approach despite the absence of universally accepted targets or monitoring intervals [148]. For mTOR inhibitors (sirolimus/everolimus), routine TDM is also recommended because of substantial pharmacokinetic variability and drug–drug interaction [149]. Although pharmacokinetics favour AUC-based monitoring over trough levels for mycophenolate, the need for multiple timed samples limits routine implementation, and targeted AUC assessment is therefore mainly reserved for situations with uncertain absorption, suspected drug–drug interactions, or during CNI transitions [150, 151]. Dried blood spot sampling of tacrolimus has shown promising feasibility and may improve convenience and patient engagement, although current evidence remains insufficient to support routine use [152, 153]. TDM is also relevant for key anti-infective agents used in LTx care. Triazole antifungals require monitoring due to highly variable exposure and potent cytochrome P4503A4-mediated interactions that increase CNI levels, antivirals such as (val)ganciclovir require renal function-guided dosing to balance efficacy and marrow toxicity, while aminoglycosides warrant level-guided dosing to maintain efficacy and limit nephrotoxicity in patients already exposed to CNI-related nephrotoxicity [154–157].

### Donor-specific antibody testing

**FIGURE 3:**
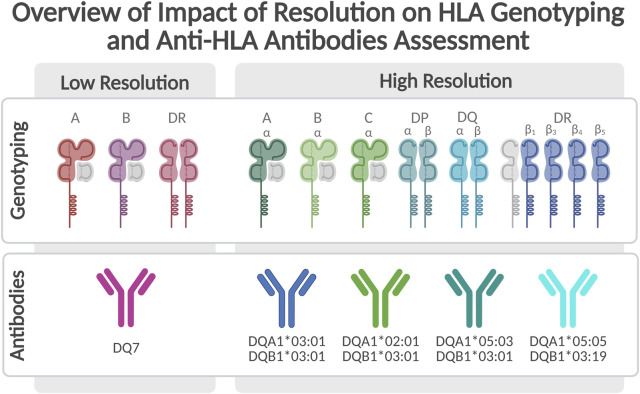
An overview of impact of resolution on HLA genotyping and anti-HLA antibodies assessment.

Evaluation of donor-specific anti-HLA antibodies remains central to diagnosing AMR [158]. Practice patterns vary widely, ranging from low-sensitivity screening assays to routine use of high-resolution single-antigen bead platforms [159–161]. Some centres incorporate scheduled surveillance, whereas others reserve testing upon clinical deterioration [160–162]. Methodological rigour is essential: kidney transplant data indicate that nearly one-quarter of donor-specific antibodies may be misclassified using lower-resolution assays, underscoring the importance of high-fidelity testing in transplant recipients [163] Figure 3. Although evidence for non-HLA antibodies in allograft injury is increasing, it remains insufficient to justify their routine use in clinical decision-making after LTx [164, 165].

### General haematological and biochemical monitoring

Comprehensive hematologic and biochemical evaluation provides valuable insight into immunologic and clinical status after LTx [2]. Leukopenia is common after LTx and has been associated with increased infectious complications and poorer clinical outcomes [166–168]. Immunophenotyping of lymphocytic subsets may help tailor immunosuppressive therapy, yet lung-transplant evidence remains scarce [169]. Eosinophilia, while nonspecific, may signal graft injury or early rejection [170]. Trends in haemoglobin and platelet counts can reveal marrow suppression, chronic disease anaemia, bleeding diatheses, or thrombotic microangiopathy [171–174].

Biochemical indices further refine clinical assessment. Elevations in C-reactive protein or procalcitonin may provide early, non-specific signals of inflammatory activity or systemic infection [175, 176]. Serum creatinine remains indispensable given well-recognised CNI nephrotoxicity, and liver enzyme abnormalities may indicate hepatotoxicity related to immunosuppressants, antifungals, or antivirals [177–180]. Broader metabolic screening is also important: dyslipidaemia is associated with post-transplant mortality, poor glycaemic control [181], and vitamin D deficiency is common and linked to higher rates of infection and adverse outcomes, and although lung-specific data are limited, routine thyroid function testing is often included to detect immunosuppression- or comorbidity-related endocrine disturbances [182–186].

From an immunologic perspective, hypogammaglobulinemia—particularly low IgG—correlates with increased infection burden, and while advanced immune phenotyping may offer additional insight into immunosuppressive intensity, current evidence does not support routine its clinical implementation [187–190].

### Microbiological surveillance

Viral monitoring is now embedded in most follow-up pathways, although practices vary between centers. CMV viral load monitoring is widely used for surveillance and early detection of breakthrough viremia [191, 192]. Ebstein-Barr virus assessment is often pursued when evaluating the risk of post-transplant lymphoproliferative disorder and may also provide indirect insight into the net state of immunosupression [193, 194]. Although best characterised in the kidney transplantation, polyomavirus infection is increasingly recognised in LTx, particularly in patients with unexplained renal dysfunction, where viremia or nephropathy should be considered [195, 196].

### Novel biomarkers

Dd-cfDNA has emerged as a promising non-invasive biomarker of allograft health, with accumulating evidence and recent consensus statements from the European Society for Organ Transplantation highlighting its sensitivity for the early detection of allograft injury, including subclinical processes that may precede overt functional decline [197]. However, dd-cfDNA remains a non-specific marker of injury, as elevated levels do not differentiate between underlying causes such as acute rejection, infection, or other forms of graft damage, thereby necessitating correlation with clinical, functional, imaging, and histopathological findings [197, 198]. While these characteristics support its role as a complementary surveillance tool, its optimal integration into routine post-LTx care—including thresholds, timing, and clinical decision pathways—as well as its cost-effectiveness, remain to be defined. Ongoing prospective studies, including the LAMBDA-001 trial, are expected to provide important real-world data to better clarify its clinical utility and inform evidence-based implementation [197–199].

Torque teno virus load has emerged as a promising biomarker of the functional state of immunosuppression in LTx recipients, while epithelial injury markers such as club cell secretory protein, circulating extracellular vesicles/exosomes carrying lung self-antigens or immune-regulatory miRNAs, and cfDNA methylation–based tissue-of-origin mapping offer complementary insights into rejection biology, however, all remain investigational and lack sufficient validation for routine clinical implementation [139, 200–204].

## Conclusion

Despite substantial advances in diagnostics and multidisciplinary care, post–LTx surveillance remains highly heterogeneous, with wide variation in organisational models, testing strategies, and integration of emerging tools. As summarised in Table 1, current follow-up practices are characterised by fragmented structures, inconsistent use of physiological, imaging, and procedural assessments, and a growing number of promising biomarkers that lack sufficient validation for routine clinical implementation. Moving the field forward will require practical, evidence-based, and consensus-driven guidance that defines minimum surveillance standards, emphasises trajectory-based interpretation of longitudinal data, and provides a clear framework for the stepwise incorporation of novel diagnostics and digital technologies into harmonised post-transplant care. Yet any meaningful standardization must acknowledge that surveillance practices are inevitably affected by geographical and logistical factors including healthcare system infrastructure, resource availability, reimbursement policies, and centre volume, which render a one-size-fits-all approach not desirable. Over-surveillance in low resource environments may divert capacity from higher-priority clinical needs which would be harmful for the overall program results. Ultimately, this highlights the critical gap where current clinical practice often reflects eminence-based rather than evidence-based decision-making; a gap that rigorous, consensus-driven standardisation can meaningfully close by establishing a minimum set of universal surveillance requirements, while offering an expanded framework of additional recommendations for higher-resourced centres with the capacity to implement them.

**TABLE 1:** Major gaps in current post–LTx surveillance, highlighting limitations of existing practices, sources of inter-centre variability, and priority areas for future standardisation and evidence generation.

Domain/Section	Current practice/Strengths	Weak spots and limitations	Unmet needs/Future directions
*Follow-Up Organisation*	Transplant pulmonologists coordinated care; structured clinics in many centres	Heterogeneous pathways; inconsistent coordination with local providers	Consensus on core organisational models; evidence linking structure to outcomes
*Documentation and Monitoring*	Electronic systems increasingly used; registries support auditing	Fragmentation between systems; inconsistent templates	Standardised templates; interoperable platforms; automatic alerts
*Adherence*	Use of BAASIS, refill data, drug levels	No gold standard; psychosocial drivers under-recognised	Validated LTx-specific tools; trials of adherence interventions
*PROMs and PREMs*	Growing use; digital tools feasible	Few validated tools; unclear clinical integration	Broader validation; guidance for real-time use
*Site of Follow-Up and Telemedicine*	Hybrid specialist–local care; telehealth expanding	No consensus on task division; variable infrastructure	Evidence-based shared-care models; telemonitoring standards
*Spirometry*	FEV_1_ and FEV_1_/FVC is cornerstone; widely accessible	% Predicted misclassification; SVC rarely measured	Transplant-specific LLN/z-scores; validation of FEV1/SVC.
*Plethysmography*	Central for CLAD phenotyping	Limited availability; difficult in severe dyspnoea	Consensus on TLC intervals; alternatives for advanced disease (e.g., imaging-derived lung volumes)
*DLCO*	Independent prognostic value; complements spirometry and phethysmography	Not in CLAD criteria; limited longitudinal data	Long-term studies; DLCO-informed longitudinal risk models
*Imaging*	CT with expiratory scans key for CLAD phenotyping; expiratory imaging valuable	No consensus intervals	Evidence-based imaging schedules; externally validated ML-enhanced CT models linked to outcomes
*Laboratory Monitoring*	TDM, DSA, viral PCR widely used	Assay variability; lack of unified thresholds	Harmonised protocols; integration of advanced biomarkers (e.g., dd-cfDNA) into longitudinal surveillance algorithms
*Bronchoscopy Protocols*	Widely used; early-phase utility clear	Survival/CLAD benefit unproven; heterogeneity high	Trials defining optimal schedules; personalised bronchoscopy intensity
*TBB*	Diagnostic standard for rejection	Low sensitivity for CLAD; interobserver variability	Standardisation; clinical validation of cryobiopsy and molecular transcriptomics
*BAL and Immunoprofiling*	Widely used for infection; consensus technique	Variable diagnostic cut-offs; cytology inconsistent	Prospective validation of BAL biomarkers; molecular signatures
*Novel Biomarkers*	Emerging biomarkers such as dd-cfDNA, transcriptomic signatures, exosomal markers, and immune profiling show promise for earlier or subclinical injury detection	Limited specificity for injury phenotype; uncertain thresholds; incomplete validation; unclear cost-effectiveness and real-world utility	Prospective validation; definition of actionable thresholds; biomarker-guided surveillance pathways; assessment of cost-benefit and implementation in routine care
*ML/Digital Analytics*	Increasing availability of multimodal digital data streams from lung function, imaging, laboratory testing, and remote monitoring creates opportunities for ML-supported surveillance	Most models remain retrospective, single-centre, and insufficiently validated; limited interpretability and uncertain clinical utility	External validation, calibration across centres, transparent reporting, and prospective trials assessing whether ML-supported surveillance improves outcomes and standardisation
*Overall Strategy*	Growing recognition of the need for multimodal, trajectory-based surveillance and increasing availability of digital data streams amenable to AI-based analysis	Absence of unified surveillance guidelines; high inter-centre variability; limited prospective validation and clinical integration of AI-driven tools	International consensus defining minimum surveillance standards, coupled with development and validation of multimodal, trajectory-based risk stratification tools incorporating AI.
